# Concentrations of ^137^Cs radiocaesium in the organs and tissues of low-dose-exposed wild Japanese monkeys

**DOI:** 10.1186/s13104-020-04972-z

**Published:** 2020-03-02

**Authors:** Toshinori Omi, Sachie Nakiri, Setsuko Nakanishi, Naomi Ishii, Taiki Uno, Fumiharu Konno, Takeshi Inagaki, Atsushi Sakamoto, Masayuki Shito, Chihiro Udagawa, Naomi Tada, Kazuhiko Ochiai, Takuya Kato, Yoshi Kawamoto, Shuichi Tsuchida, Shin-ichi Hayama

**Affiliations:** 1 Nippon Veterinary and Life Science University, 1-7-1 Kyonancho, Musashino City, Tokyo 180-8602 Japan; 2 Conservation and Animal Welfare, 1-9-4 Kunitachi City, Tokyo, 186-0004 Japan; 3 Fukushima-Mirai Agricultural Cooperative, 19-2 Kubota, Kamata, Fukushima, Fukushima 960-0102 Japan; 4 Jichi Medical University, School of Medicine, 3311-1 Yakushiji, Shimotsuke City, Tochigi 329-0498 Japan

**Keywords:** Japanese monkeys, Fukushima Daiichi Nuclear disaster, Caesium, Radioactive contamination

## Abstract

**Objectives:**

Following the massive earthquake that struck eastern Japan on March 11, 2011, a large amount of radioactive material was released into the environment from the damaged reactor of the Fukushima Daiichi Nuclear Power Plant (FDNPP). After the FDNPP accident, radiocaesium was first detected in muscle samples from wild Japanese monkeys exposed to radioactive materials, and haematologic effects, changes in head size, and delayed body weight gain were also reported, but little is known about the distribution of ^137^Cs in the organs and tissues of wild Japanese monkeys.

**Results:**

We detected the ^137^Cs in various organ and tissue samples of 10 wild Japanese monkeys inhabiting the forested areas of Fukushima City that were captured between July and August 2012. Among muscle, brain, heart, kidney, liver, lung, and spleen, muscle exhibited the highest and the brain the lowest ^137^Cs concentration. The concentration (mean ± SD) of ^137^Cs in muscle, brain, heart, kidney, liver, lung, and spleen was 77 ± 66, 26 ± 22, 41 ± 35, 49 ± 41, 41 ± 38, 53 ± 41, and 53 ± 51 Bq/kg, respectively. These results can help us understand the biological effects of long-term internal radiation exposure in non-human primates.

## Introduction

Following the massive earthquake that struck eastern Japan on March 11, 2011, a large amount of radioactive material was released into the environment from the damaged reactor of the Fukushima Daiichi Nuclear Power Plant (FDNPP) [1–4]. To date, the effects of exposure to the radioactive materials released from the FDNPP have been investigated not only in humans [5, 6] but also in blue butterflies [7], cattle [8, 9], monkeys [10–13], fish [14], birds [15], pigs [16], and wild boar [17].

The Japanese monkey, one of the world’s northernmost wild primates, is endemic to Japan and has a life span of more than 20 years [18]. Japanese monkeys usually form troops consisting of 50–100 individuals of one maternal lineage, and each troop occupies a home range spanning approximately 4–27 km^2^ in snowy areas [19–21]. The monkeys feed primarily on plant leaves and fruits but also eat insects and other small animals [22]. We began a research project in 2011 to examine the health effects of long-term radiation exposure on wild Japanese monkeys inhabiting the forested areas surrounding Fukushima City, which is located 70 km from the FDNPP, and we anticipate that our findings will provide valuable data and insights. In addition, data from non-human primates—the closest taxonomic relatives to humans—should make a notable contribution to future research examining the health effects of radiation exposure in humans. In our previous study, we detected high levels of radiocaesium in muscle [10] and reported haematologic effects [11] as well as changes in head size and delayed body weight gain in wild Japanese monkeys [12] after the FDNPP disaster. In the present study, we determined the distribution of ^137^Cs in various organs and tissues of wild Japanese monkeys inhabiting the forested areas surrounding Fukushima City that were captured between July and August 2012.

## Main text

### Materials and methods

#### Animals

Samples of muscle, brain, heart, kidney, liver, lung, and spleen were collected from 10 Japanese monkeys (*Macaca fuscata*) captured between July and August 2012 from the forested areas around Fukushima City. These monkeys were used in a previously reported study to determine the concentrations of ^134^Cs and ^137^Cs in the muscles and characterize the changes in concentration over time as well as their relationship with soil contamination levels [12]. Organ and tissue samples were stored frozen at − 30 °C after 2012 until they were used in the present study. The age of each animal was estimated from the status of tooth eruption, as described by Iwamoto et al. [23], to divide the animals into the following age groups: juveniles (0–3 years), subadults (4–5 years), and adults (≥ 6 years).

#### Radioactivity measurements

Samples of skeletal muscle, brain, heart, kidney, liver, lung, and spleen were analysed using a germanium semiconductor spectrometer (Canberra, GC2020-7500SL-2002CSL, Meriden, CT) in Food Allergy Research Laboratories FARL (Maebashi, Gunma, Japan). Data were corrected to the background radiation dose of the measurement environment on an as-needed basis. ^137^Cs was detected based on 661.6-keV gamma-ray energy. Collected samples were stored frozen at − 30 °C until radioactivity measurements. This method of measurement is the same as that used in our previous study [12]. The radiocaesium radioactivity level was adjusted to the value on the day of capture based on physical half-life. The limit of detection was 5 Bq/kg.

#### Statistical analysis

Differences in mean ^137^Cs concentration between groups were analysed by one-way ANOVA using SPSS, ver. 19 (IBM Corp., Armonk, NY, USA).

### Results and discussion

**Fig. 1:**
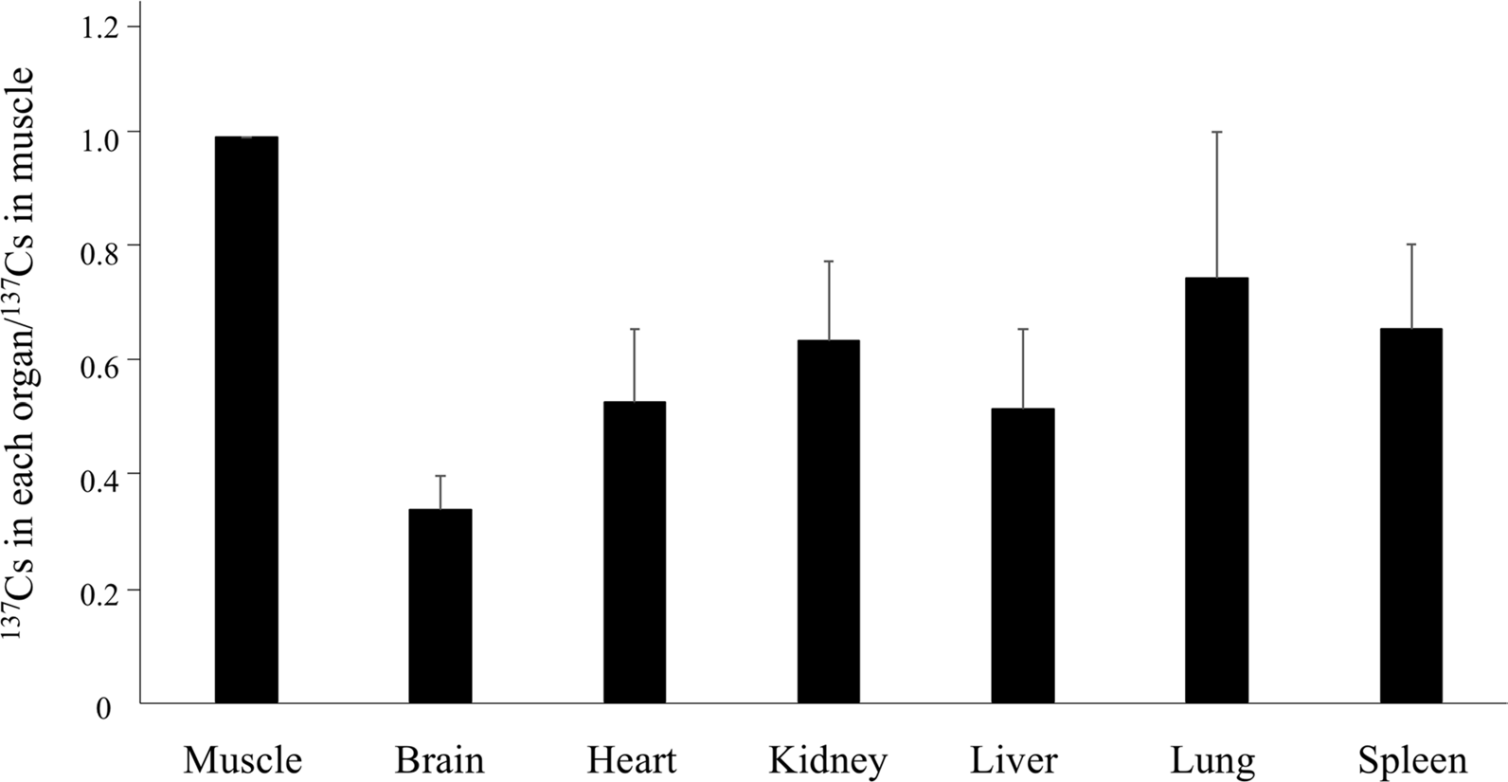
Distribution of ^137^Cs in organ and tissue samples collected from 10 Japanese monkeys in 2012. Graph shows the ratio of the concentration of ^137^Cs in each organ to that in muscle (^137^Cs in each organ/^137^Cs in muscle). Error bars indicate SD

Table 1 and Fig. 1 show the concentrations of ^137^Cs in skeletal muscle and various organ samples obtained from 10 Japanese monkeys captured between July and August 2012. The monkeys inhabited the forested areas surrounding Fukushima City, which is located 70 km from the FDNPP. The average ^137^Cs concentration in the skeletal muscles and internal organs ranged from 26 to 77 Bq/kg. Among the skeletal muscle, brain, heart, kidney, liver, lung, and spleen, muscle exhibited the highest and the brain the lowest ^137^Cs concentration. The mean (± SD) ^137^Cs concentration in the muscle, brain, heart, kidney, liver, lung, and spleen was 77 ± 66, 26 ± 22, 41 ± 35, 49 ± 41, 41 ± 38, 53 ± 41, and 53 ± 51 Bq/kg, respectively. There were no significant differences in mean ^137^Cs concentration between groups, as determined by one-way ANOVA. The ratio of the concentration of ^137^Cs in each organ to that in muscle (^137^Cs in each organ/^137^Cs in muscle) was 0.34 ± 0.06, 0.53 ± 0.13, 0.64 ± 0.14, 0.52 ± 0.14, 0.70 ± 0.26, and 0.66 ± 0.15 for the brain, heart, kidney, liver, lung, and spleen, respectively.

**Table 1:** Concentration of ^137^Cs in various organs and tissues of Japanese wild monkeys

Lab.ID	Date of capture	Sex	Age	Cs-137(Bq/kg)							
				Muscle	Brain	Heart	Kidney	Liver	Lung	Spleen	Soil
FF-890	2012/7/10	Male	0–3	207	67	114	126	120	132	142	318,233
FF-895	2012/7/17	Male	≥ 6	53	22	19	46	25	35	27	78,473
FF-897	2012/7/21	Male	0–3	41	18	26	28	28	25	27	n.t
FF-898	2012/7/21	Female	0–3	51	21	40	39	39	36	48	n.t
FF-900	2012/7/23	Female	0–3	38	13	21	25	19	26	24	74,236
FF-902	2012/7/26	Female	4–5	33	8.5	15	16	13	31	15	74,236
FF-904	2012/7/27	Female	0–3	194	68	97	124	101	127	156	220,296
FF-915	2012/8/7	Male	0–3	39	10	18	29	21	55	29	47,099
FF-916	2012/8/7	Male	0–3	66	19	39	37	18	40	39	47,099
FF-925	2012/8/26	Male	≥ 6	49	16	18	18	25	27	26	47,099
Mean ± SD				77 ±66	26 ± 22	41 ± 35	49 ± 41	41 ± 38	53 ± 41	53 ± 51

In this study, we found the highest concentration of radiocaesium in the skeletal muscle and lowest concentration in the brain of Japanese wild monkeys inhabiting forested areas surrounding Fukushima City. These findings agree with those of previous studies in cattle [8, 9] and wild boar [17]. Similar results in wild Japanese monkeys were recently reported by Urushibara et al. [26]. However, the order of the relative ^137^Cs concentration of the internal organs seemed to be slightly different between species. In particular, the lung and spleen showed high ^137^Cs concentrations in Japanese monkeys.

The muscle radiocaesium concentration in the monkeys correlated significantly with the level of soil contamination at each capture location [12]. The ^137^Cs concentration in organs of two individuals, labelled FF-890 and FF-904, was higher than that in other individuals, which was related to the soil concentration of radiocaesium.

These data regarding the distribution of ^137^Cs in the organs and tissues of Japanese monkeys captured in the areas surrounding Fukushima City will enhance our understanding of the biological effects of long-term internal radiation exposure. Continued monitoring of Japanese wild monkeys exposed to radioactive materials following the FDNPP accident would help in understanding the biological effects of long-term internal radiation exposure to the natural world.

## Limitations

Continued monitoring of Japanese wild monkeys exposed to radioactive materials following the FDNPP accident would help in understanding the variation of the ^137^Cs concentration among the organs of the body and its biological effects under long-term internal exposure.

## Data Availability

Only publicly available data were analysed in this paper. The final datasets and code are available from the corresponding author upon request.

## Abbreviations

FDNPP: Fukushima Daiichi Nuclear Power Plant
Cs: Caesium
ANOVA: Analysis of variance
